# Oropharyngeal HPV infection: prevalence and sampling methods among HIV-infected men in South Africa

**DOI:** 10.1177/0956462418755882

**Published:** 2018-02-22

**Authors:** Admire Chikandiwa, Pedro T Pisa, Matthew F Chersich, Etienne E Muller, Philippe Mayaud, Sinead Delany-Moretlwe

**Affiliations:** 1 Wits RHI, Faculty of Health Sciences, University of the Witwatersrand, Johannesburg, South Africa; 2 National Institute for Communicable Diseases, National Health Laboratory Service, Johannesburg, South Africa; 3 London School of Hygiene and Tropical Medicine, London, UK

**Keywords:** Human papillomavirus, oral sex, oral cavity, men, Africa

## Abstract

Worldwide, 96,000 cases of oropharyngeal cancer (OPC) occurred in 2012. Human papillomavirus (HPV) is a risk factor for OPC. Data on oropharyngeal HPV infection are limited. There is no consensus on the best sampling method for detecting the infection. We describe the prevalence of oropharyngeal HPV infection among HIV-infected men and compare the performance of oral rinses and swabs in detecting oropharyngeal HPV infection. Paired oral rinses and swabs for 181 men were tested for HPV DNA using the Roche Linear Array. Performance was determined by the number of infections detected and the percentage of samples with adequate DNA extraction. Agreement between sampling methods was assessed by the kappa statistic. Prevalence of oropharyngeal HPV infection with rinse samples was 1.8% (three infections) and 0.6% (one infection) with swabs (p = 0.06). Adequate cellular DNA extraction was more likely with rinse (93.4%) than swab samples (89.0%, p = 0.05). There was moderate agreement between the methods (kappa = 0.49). The prevalence of oropharyngeal HPV DNA infection among this predominantly heterosexual sample of men living with HIV was low and consistent with the infrequent oral sex practices. Oral rinse performed better than oral swab in detecting oropharyngeal HPV DNA infection and might contribute to screening for OPCs.

## Introduction

Worldwide, an estimated 96,000 cases of oropharyngeal cancer (OPC) occurred in 2012,^1^ with incidence rising over time.^2^ The rate of OPCs is about 3–5-fold higher in men than in women.^2^ A similar higher rate of OPC in men than in women was also reported in South Africa.^3^

Human papillomavirus (HPV) plays an aetiological role in OPCs, with 25–50% of OPCs related to HPV.^1,2^ Oropharyngeal HPV infections mirror the gender patterns of OPCs.^4^ Higher oropharyngeal HPV prevalence in men has been ascribed to men being more likely to smoke, with smoking interfering with mucosal immunity, raising susceptibility to HPV infection. Women are more likely to generate antibodies following genital HPV infection than men, with these antibodies offering some protection against oral HPV infection.^4^ In addition, women have higher genital HPV viral loads, making transmission higher with male–female oral sex than female–male.^4^ HIV-infected men have a higher risk for oropharyngeal HPV infection and OPCs than HIV-negative men.^5,6^

Data on prevalence of oropharyngeal HPV infection from low- and middle-income countries are limited. The prevalence among healthy men in Brazil was reported as 2%.^7^ A study in Pretoria, South Africa documented a prevalence of 6% among 125 men, of whom 4% were HIV infected.^8^ Another study of 34 men in Soweto, South Africa, of whom 9% were HIV infected, reported a prevalence of 18%.^9^ It is difficult to directly compare prevalence estimates, as sexual orientation, HIV sero-status, sample sizes and HPV sampling methods vary between studies. Using baseline data from a cohort of men living with HIV infection (MLWH) in Johannesburg, South Africa, we document the prevalence of oropharyngeal HPV and compare the performance of oral rinses and oral swabs in detecting these infections.

## Methods

The cohort study enrolled 304 men, of whom 181 were randomly selected for this sub-study using random numbers generated in Microsoft Excel. Full details of study procedures are described elsewhere.^10^ Briefly, men were enrolled if 18 years or older, HIV-positive and reported sex in the past three months.^10^ We collected socio-demographic and behavioural data using a questionnaire. Participants completed sensitive questions on sexual behaviour using computer-assisted self-interview (CASI). The primary aim of the cohort study was to evaluate the natural history of HPV infection and disease in HIV-infected men in South Africa to help inform the selection of HPV prevention interventions in this population. The sample size of 181 was deemed adequate to compare the performance of oral rinse and oral swabs with sensitivity of 70%, specificity of 90% and precision of 10%. Calculations were based on oral HPV prevalence of 20%. We assumed that the prevalence of oropharyngeal HPV infection in men who were all HIV-positive would be higher than the 18% in men in nearby Soweto, of whom only 9% were HIV-positive.^9^ The study was approved by Wits Human Research Ethics Committee (Approval number: M111191). Written, informed consent was obtained from participants after explanation of study objectives and procedures.

A comprehensive oral examination was conducted by medical officers, which included inspection of the gums, tonsillar pillars, tonsils, retropharyngeal wall and palate. Oral rinses were first collected by asking participants to gargle 15 mL of normal saline for 15 s before spitting the fluid into a container and centrifuging it at 3000 r/min for 10 min to obtain sediment. A cotton swab was then rubbed against the palatine tonsil and oropharynx. At the same visit, to assess anogenital-oropharyngeal HPV genotypic concordance, a genital swab was collected by rubbing a cotton swab around the glans penis, coronal sulcus and ventral surface of the penis. Intra-anal swabs were collected by blindly inserting a Dacron swab 3 cm into the anal canal and removing it while rotating and applying pressure on the walls of the canal. The swabs and sediment were stored at −70°C before HPV DNA testing. Venous blood was collected for CD4 cell count and HIV-1 plasma viral load (PVL) testing.

The MagNA Pure LC DNA Isolation Kit I (Roche Diagnostics, Mannheim, Germany) was used to extract HPV DNA from the swabs and sediment. HPV genotype distributions were assessed by the Roche Linear Array assay (Roche Diagnostics, Mannheim, Germany). The human β-globin gene served as an internal control for cellular adequacy, extraction efficiency and amplification. Therefore, if the internal control is positive, then HPV will be detected if present. All HPV test strips were interpreted separately by two people to minimise reading errors.

### Statistical analysis

Fisher’s exact test was used to compare the percentages of samples with adequate cellular DNA extraction and HPV DNA positive as well as characteristics of participants with and without oropharyngeal HPV infection. Agreement between the two sampling methods was assessed by the percentage of crude agreement, defined as p = (*a + d*)/N, where *a* = number positive by both assays, *d* = number negative by both assays and N = total sample and the kappa statistic. Since the contingency table was unbalanced, we calculated percentage positive and negative agreements, defined as *a*/[0.5 × (N+ *a*−*d*)] and *d*/[0.5 × (N − *a* + *d*)], respectively.^11^ Stata™ Version 13 was used for all analyses.

## Results

The median age was 39 years, with only 2% aged under 25 years (age range: 23–62). More than one-fourth smoked (28%) and half drank alcohol (53%). Eighty per cent were taking antiretroviral therapy (ART), of whom 55% had undetectable PVL. The median number of life-time sexual partners was 30, with 27% reporting more than 100 partners. A quarter had had more than one sexual partner in the preceding three months (27%). Smaller proportions reported ever having oral sex: 15% oral-genital and 4% oral-anal contact. Only 7% reported ever having sex with men (MSM).

Oral rinse samples were more likely to have adequate cellular DNA extraction than the oral swab samples (169/181 [93%] vs. 161/181 [89%]; p = 0.05). Oral rinse samples identified three HPV infections, compared to only one with oral swabs (p = 0.06). Overall, 82% of the samples gave concordant results, kappa = 0.09, indicating minimal agreement. When analysis was restricted to the 151 samples with adequate DNA extraction, there was moderate agreement between the sampling methods (99%, kappa = 0.49). Corresponding percentages of positive and negative agreement were 50% and 99%, respectively (Table 1).

**Table 1.** Agreement between oral rinse and swab sample for HPV DNA detection among 181 male HIV-1 seropositive participants in Johannesburg, South Africa.

	Oral rinse +	Oral rinse −	Crude agreement (%)	kappa	Positive agreement (%)	Negative agreement (%)
All samples^a^ (n=181)
Oral swab +	1	0	82	0.09	6	90
Oral swab −	2	148				
Samples with adequate DNA (N=151)^b^			
Oral swab +	1	0	99	0.49	50	99
Oral swab −	2	148				

^a^Includes 30 samples that had inadequate cellular DNA extraction as measured by the β-globin gene (18 oral swabs; 8 oral rinses; 2 on both oral rinse and oral swab).

^b^Includes only samples that had adequate cellular DNA extraction on both oral swab and rinse.

Overall, prevalence of HPV DNA was 1.8% (95% confidence interval: 0.4–5.1%; 3/169). The three participants with oropharyngeal infections had high-risk sexual behaviour. They all reported a sexual debut aged under 18 years, more than one sexual partner in the past three months and had not used a condom with their most recent partner. They were all clinically stable: on ART for more than 18 months, with CD4 cell count above 500 cells/µL and PVL less than 50 copies/mL. No macroscopic oropharyngeal lesions were found on examination of these three participants (Table 2).

**Table 2.** Attributes of three participants with oropharyngeal HPV infection.

Characteristic	Participant A	Participant B	Participant C
Age (years)	44	39	40
Marital status	Single	Single	Single
Currently smokes	No	No	No
Currently drinks alcohol	Yes	No	Yes
Age at sexual debut	17	10	17
Number of sexual partner in past three months	6	15	2
Ever had oral-genital contact^a^	Yes	Yes	No
Ever had oral-anal contact	No	No	No
Ever had sex with men	No	Yes	No
Consistent condom use with recent partner	No	No	No
Taking ART	Yes	Yes	Yes
Duration on ART (months)	93	54	19
CD4+ cell count, cells/µL	751	655	525
HIV-1 PVL (copies/mL)	<40	41	48
Oropharyngeal HPV types isolated	HPV 72	HPV CP6108	HPV 72
Genital HPV types isolated	HPV 72	HPV CP6108	HPV 45 & HPV 70
Anal HPV types isolated	None	HPV CP6108	None
Macroscopic oral or oropharyngeal lesion	No	No	No

PVL: plasma viral load; ART: antiretroviral treatment; HPV: human papillomavirus.

^a^Oral-genital refers to oro-penile and oro-vaginal contact.

We also examined anal and genital samples of the three men who had oropharyngeal HPV infection (Table 2). Participant A reported oral-genital contact and had the same HPV genotype 72 isolated from oral rinse, oral swab and genital swab, but no infection on anal swab. Participant B reported sex with other men and oral-genital contact and the same HPV CP6108 genotype isolated on oral rinse, genital swab and anal swab samples. Lastly, Participant C did not report oral sex; the HPV genotype 72 isolated from the oral rinse differed from the genital HPV genotypes (45, 70), and anal swab was negative.

## Discussion

Our study shows that oropharyngeal HPV prevalence in this population is low and that the detection of infection is influenced by sampling method. The prevalence of infection was similar to the 2% reported among men in Brazil,^7^ but is several fold lower than the two previous estimates from South Africa.^8,9^ These variations likely reflect differences in sexual behaviours between the study populations since all three studies used oral rinse samples. For example, 80% of men in the Soweto study reported oral sex compared to only 15% in our study.^5,9^ Men in our study reported higher numbers of lifetime sexual partners than the average (10) documented for Southern Africa.^12^ The reason is unclear, but CASI compared to interviewer-administered questionnaire (used in the Pretoria study) and self-administered questionnaire (used in the Soweto study) could have minimised social desirability bias. This is plausible because being an MSM is still stigmatised within South African communities despite the decriminalisation of homosexuality in 1994.^13^

We found concordant HPV DNA genotypes from anogenital and oral samples in two participants who reported oral sex. This supports the contention that oral sex is responsible for anogenital to oropharyngeal transmission of HPV infection, and that the observed rise in OPC associated with HPV may be attributed to changes in sexual practices.^2^ This is in keeping with our finding that the anal or genital HPV infection status per se was not associated with oropharyngeal infection. However, HPV auto-inoculation through non-sexual means, such as contaminated fomites or fingers is possible.^14^ It is noteworthy that no HPV type 16 infection, which is mainly implicated in OPC, was isolated. The implications of this finding are unclear but could relate to the generally low prevalence of oropharyngeal HPV infection among these men who all had no OPC. Future studies which test the HPV status of histologically-confirmed OPC cases in South Africa are required to better understand the role of HPV type 16 in the causation of these tumours.

Similar to previous studies, we found that oral rinsing is better than oral swabbing for ensuring adequate cellular DNA extraction and detecting oropharyngeal HPV infection.^15^ This is likely due to the rinse washing out exfoliated cells from around the oral cavity, while oral swabs may be unable to access areas like the tonsillar crypts.^15^ Overall, there was minimal–moderate agreement between the two sampling methods. The difference between the high crude agreement and moderate agreement from kappa is explained by the percentage positive and negative agreements which show that the main area of disagreement between the sampling methods was in positive results.^11^

The main limitations of this study are that it is cross-sectional and the prevalence of oropharyngeal HPV infection is low, making it difficult to detect associations. Additionally, it consisted of mainly heterosexual HIV-positive men, and findings may not be extrapolated to other populations. Concordance of genotypes from anogenital and oral samples does not necessarily imply that these are the identical viruses and it is possible that these were two separate infections with the same HPV type since viral gene sequencing was not done. Despite these limitations, it contributes literature on the prevalence of oropharyngeal HPV infection among men in South Africa and informs decisions about oropharyngeal sampling.

## Conclusion

The prevalence of oropharyngeal infection among this predominantly heterosexual sample of MLWH was low and consistent with the infrequent oral sex practices. Oral rinse performed better than oral swab in detecting oropharyngeal HPV DNA infection and might contribute to screening for OPCs.

## Key messages

- Prevalence of oropharyngeal infection among this predominantly heterosexual sample HIV-positive is low.
- Oral sex is a risk factor for oropharyngeal HPV infection.
- Oral rinse performed better in detecting oropharyngeal HPV DNA infection and might contribute to screening for OPCs.

## Availability of data and materials

The dataset generated during and/or analysed during the current study is not publicly available as it contains longitudinal data which are yet to be published but are available from the corresponding author on reasonable request.
